# Hydrology for impact: building partnerships, blending knowledge and bracing for climate change

**DOI:** 10.1098/rsta.2024.0290

**Published:** 2025-07-31

**Authors:** Harriet G. Orr, Caitlyn A. Hall, Vicki Rhodes, Rob L. Wilby, Katy L. Peat, Hayley J. Fowler

**Affiliations:** ^1^ Environment Agency, Horizon House, Bristol, UK; ^2^ Biosystems Engineering Department, The University of Arizona College of Agriculture and Life Sciences, Tucson, AZ, USA; ^3^ Department of Geography and Environment, Loughborough University, Loughborough, Leicestershire LE11 3TU, UK; ^4^ Department for the Environmnet Food and Rural Affairs, London, UK; ^5^ School of Engineering and Tyndall Centre for Climate Change Research, Newcastle University, Newcastle upon Tyne NE1 7RU, UK

**Keywords:** hydrology, research impact, knowledge, participatory, co-production, multi-disciplinary

## Abstract

The hydrology community plays a critical role in understanding, communicating and managing hydroclimatic hazards and water security. As climate-related trends and risks emerge, there is an urgent need to help communities and organizations prepare for changes that are already underway and expected to become more severe. There is consensus about the need for transdisciplinary collaboration and participatory research to co-create knowledge to support informed decision-making. However, there is less clarity about how this should be done in practice. Achieving meaningful societal impact through research is not an exact science, and we do not propose a definitive framework or ‘recipe for success’. Instead, we reflect on our collective experiences over the last 20 years and surmise that strong partnerships, open communication and a willingness to embrace uncertainty can accelerate the impact of hydrological research on policy and practice and hence societal preparedness for climate change. We also advocate the development of new metrics—beyond research income and citations—to incentivize academics to plan for, and engage in, more impactful research-into-practice. We further call on government departments, research funders, professional bodies, societies and business associations to support the enabling environments needed to achieve this outcome.

This article is part of the Royal Society Science+ meeting issue ‘Hydrology in the 21st century: challenges in science, to policy and practice’.

## 1. Hydrology’s role in a changing climate: framing the challenge

Hydrological research plays a critical role in addressing the accelerating impacts of human activity on ecosystems and the growing challenges of ensuring sufficient water, food and energy in a changing climate. Global hydrological risks—such as extreme flooding, droughts, storm surges and sea level rise—are well-documented indicators of the rising threats to water availability and quality [1–3].

Research has progressed our understanding of climate-related water challenges [4]. However, the functioning of hydrological systems—including water resources and water quality—is shaped not only by environmental processes but also by socio-economic and political structures that influence how resources are allocated, which policies are prioritized and who participates in decision-making [5,6].

Socio-political systems and policy frameworks tend to respond slowly to new research, creating lags between scientific discovery and translation into actionable strategies. Despite the growing body of evidence that climate change is exacerbating hydrological risks, policies and infrastructure are not evolving quickly enough to reflect the scale and speed of these changes. Limited use of scientific knowledge is just one challenge in the context of a range of institutional, informational and systems barriers. However, this disconnect is often exacerbated by short-term political cycles, competing economic interests, bureaucratic inertia and lack of resources—all of which can delay implementation of research-driven solutions [2].

Research, funding and working within disciplinary silos often reinforce barriers by prioritizing short-term, discipline-specific outputs (e.g. publications and metrics) over long-term, cross-sectoral work needed to address systemic water challenges [7]. Consequently, hydrological research is frequently disconnected from the governance contexts where decisions are made and from the communities affected [5,6]. Hydrology remains largely supply-driven by researchers, shaped by disciplinary agendas rather than the needs of those managing or experiencing water-related risks. Disciplinary silos and misaligned timescales can result in findings that arrive too late, are viewed as too uncertain or fail to align with the framings used by policymakers and practitioners [2,8]. These issues contribute to a persistent ‘science-policy-practice gap’, where evidence is underused—not due to poor quality, but because it is mistimed, misaligned or developed without sustained trust or engagement [9–11]. In some cases, institutional mandates and rigid governance frameworks further constrain the uptake of adaptive or integrated approaches, despite a strong evidence base.

Many of us became water scientists to advance change: to contribute to the transformative adaptations needed to meet climate challenges. But it is worth asking: are our current ways of working enabling transformations critical for actionable change? Transdisciplinary and participatory approaches offer a more direct path to application-driven research and societal impact. By engaging affected communities, practitioners and decision-makers throughout the research process, we can better align hydrological research with broader societal goals for equitable and sustainable water management [12]. This shift requires moving beyond disciplinary silos and conventional academic incentives towards more collaborative work that may be less linear but ultimately more enduring [8,13].

Grand hydrological challenges and future water threats have been set out, and national and global agendas increasingly call for co-designed, action-oriented solutions to solve these [14]. In this paper, we agree that hydrology needs to move beyond a knowledge-generation paradigm towards action-oriented, participatory and transdisciplinary research. To support this, we:

— Consider essential attributes of collaboration and participatory research;
— Explore what is involved in building partnerships and understanding decision contexts;
— Describe how knowledge exchange and co-creation lead to more action and better decisions;
— Reflect on some case studies of co-developed hydrological research;
— Consider ways to navigate ideological, institutional and resource constraints that hinder progress;
— Present a future focus for hydrological research that closes the gap between knowledge and impact by building adaptive capacity through equitable partnerships.

While much of what we describe is relevant to a variety of settings, this paper draws on the collective experience of the authors across policy, regulatory and operational water management practice and with academia. Local community engagement and participation are clearly a legitimate locus for hydrological research, but are not the focus of this paper.

## 2. Collaborative pathways: positionality, leverage and impact

Effective collaboration begins with recognizing our own positionality as water researchers: how our professional and personal backgrounds influence the questions we ask, what methods we use and how we engage with others [15]. Recognizing that knowledge is not value-free [11] helps build trust, foster understanding across perspectives and lay the foundation for strong, inclusive partnerships [16]. Actively seeking complementary expertise—particularly from other disciplines, sectors and communities—helps bridge the gap between scientific insight and practical application.

As hydrologists, we are well-placed to respond to urgent water security and climate adaptation challenges. But achieving meaningful impact requires crossing disciplinary boundaries and co-creating pathways that bring together science, policy, practice and lived experience. Maximizing the uptake of research depends on building relationships across these domains to capture diverse knowledge and translate it into context-specific, actionable (practical) solutions. Through collaboration, we can address the root causes of water and climate challenges—not just their symptoms—and help build the adaptive capacity needed for an increasingly uncertain future.

To influence policy and practice, research should align with current decision-making systems, consider institutional needs and constraints, such as the remit of an organization, the window of opportunity to make changes and engage early and consistently with diverse actors. Tailoring our communication to policy cycles and making findings accessible to decision-makers and practitioners, while emphasizing their relevance, can enhance uptake. Knowledge brokers, such as science translators or boundary-crossing consultants, can support this process [17]. Participating in forums, advisory committees and legislative hearings enables co-developed questions with stronger impact potential. Programmes like the UK Parliamentary Office of Science and Technology and Regional Partnerships, such as Adaptation Scotland, provide mechanisms for engagement at various levels, as do organizations like Natural Resources Wales which actively support researcher collaboration.

Beyond government and policy, partnerships with communities are essential for producing knowledge that reflects lived realities and supports equitable outcomes. This requires active listening, transparent communication and rejecting one-size-fits-all solutions. Although resource-intensive, working with local organizations and community leaders enables the co-design of technically robust and socially fair projects [18]. For example, the UK Flood and Coastal Resilience Innovation Programme [19] funded 25 local projects aimed at enhancing resilience through practical, community-centred action. One project focused on understanding and mitigating highly localized flooding around Eastbourne, capturing the impact of changing water levels on people who use green and blue spaces and their hopes and concerns for the future [20].

Likewise, industries reliant on consumptive water use—such as agriculture, energy, manufacturing and construction—are key actors in sustainable water governance. Collaborating with industry requires understanding their operational needs and regulatory contexts while demonstrating the long-term value of sustainable, adaptive practices. Through formal joint research projects, water researchers can deliver data-driven solutions that improve efficiency, reduce environmental impacts and manage risks from droughts and floods (e.g. [21]). These partnerships position researchers as both technical advisors and advocates, helping industry align with sustainability goals and build resilience over time.

Ultimately, building adaptive capacity to address hydrological risks demands interdisciplinary partnerships that span ecological, social and economic systems. Hydrological research that goes beyond generating evidence can support implementation by engaging with policy, practice and decision-making processes [22–24]. Transdisciplinary co-production frameworks can formalize collaboration across communities, policy spaces and industries, strengthening societal resilience to climate change [25,26]. The next section explores how knowledge translation and co-creation can guide research towards more meaningful, real-world outcomes.

## 3. Knowledge sharing and co-creation approaches to actionable knowledge

Turning knowledge into action requires deliberate, sustained and participatory approaches across the full knowledge management lifecycle, from knowledge production, organization and storage to sharing, translation and use [27–29]. Yet researchers, practitioners, policymakers and communities are often treated as separate actors, reinforcing power imbalances, marginalizing lived experience and limiting shared ownership. These dynamics can entrench top-down, supply-driven approaches and reduce the usability of scientific knowledge.

Knowledge exchange offers a critical starting point to disrupt this pattern. Activities such as workshops, dialogue events and collaborative platforms can build visibility and trust while fostering mutual understanding. However, without sustained and embedded engagement, these efforts risk becoming simply transactional. Effective knowledge exchange needs to be well-resourced and supported by mechanisms for ongoing, multidirectional learning. Experiences from health and social sciences highlight the importance of trusted intermediaries—such as knowledge brokers, boundary organizations (those than span the gap between policy and implementation) and iterative frameworks—for facilitating this exchange (table 1).

**Table 1.** Example frameworks for knowledge exchange and development.

framework/methodology	objective
integrated water resources management (IWRM) [30]	promotes coordinated development and management of water, land and related resources. balances social, economic and environmental objectives.
adaptive management [31]	emphasizes learning and adapting through iterative decision-making processes. allows for flexibility in responding to changing conditions and new information.
social-ecological systems (SES) framework [32]	examines the interactions between social and ecological systems. focuses on resilience, sustainability and adaptive capacity.
knowledge-to-action (K2A) framework [33]	guides the translation of research findings into practical applications. involves identifying knowledge gaps, developing targeted strategies and implementing solutions.
triple helix model [34]	describes the interactions between academia, industry and government in fostering innovation. encourages collaboration for sustainable water management solutions.
systems thinking: theory of change [35]	shows how and why a particular action or policy will lead to a specific outcome. helps to protect and understand the socio-economic and natural systems that are affected by policy decisions.

However, knowledge exchange alone is insufficient. Lasting impact requires a deeper commitment to co-creation, where research questions, methods and outputs are developed collaboratively from the outset. Co-production helps overcome persistent barriers, including fragmented information systems, mistrust, power imbalances and institutional rigidity [9]. Embedding collaboration throughout the research cycle enables outcomes that are more context-specific, relevant and actionable [11,28,36].

Co-creation is a transformative process that shifts the role of researchers from knowledge providers to equal partners in joint knowledge development [37]. This approach fosters trust, enhances relevance and improves the likelihood of uptake [38]. When all actors are engaged as peers, the resulting knowledge reflects local expertise, meets institutional needs and acknowledges the cultural, political and historical context of water governance [13,24,38,39].

Hydrology is especially well-placed to benefit from co-creation as it is embedded within complex socio-environmental systems. Participatory modelling illustrates how sustained engagement improves problem framing, reveals system interdependencies and generates technically sound and socially acceptable solutions [40,41]. Co-creation also enables decision-making under uncertainty by valuing local context, flexibility and feedback loops.

Crucially, co-creation strengthens the flexibility of knowledge systems. In a changing climate, static solutions are increasingly inadequate. Iterative, co-created approaches ensure that interventions evolve with shifting environmental and social conditions. Long-term relationships and embedded collaboration support learning across time and space scales [42]. Hydrology’s greatest impact on immediate real-world challenges will come not just from generating or disseminating knowledge but from co-creating solutions that are grounded in context, shaped by diverse expertise and owned by those who will need to act. This is both a practical imperative for climate adaptation and a necessary step towards fairer and more effective water governance. Co-produced research generally follows an iterative process of identifying participants and building partnerships, co-exploring decision needs, co-developing research, co-delivering solutions and evaluating outcomes [29,42,43].

## 4. Learning from co-creation: successes and challenges in applied hydrology

This section draws on tools and case-based insights to illustrate how co-creation unfolds in practice within hydrology. We highlight both successes and limitations, emphasizing that co-production is not the end goal but rather the beginning of a longer arc of collaborative solution delivery. Moving from co-created research to real-world application demands sustained relationships, mutual learning and institutional capacity to evolve over time [44]. Evaluating success requires more than checking for uptake; it involves asking whether these partnerships lead to more resilient systems, less vulnerability and more capacity to adapt.

### (a) Building partnerships and exploring decision needs

Identifying the right partners is a foundational step in co-created research. This often requires mapping exercises to determine who has an interest in the issue, who holds power to effect change and—perhaps less obviously—who may be affected by or act as a barrier to change [45]. It is equally important to consider the strength of partner interests, their capacity to engage and the potential influence they may have on the research or its intended outcomes.

Given that different actors bring diverse perspectives, recognizing individual and organizational positionality is essential. This includes reflecting on how our professional and personal backgrounds shape the way we frame problems, define research questions and choose who to work with [46]. These choices may influence whether a top-down or bottom-up approach is taken and inform the kinds of solutions that emerge. Co-defining decision needs is inherently subjective: the process and outcomes depend heavily on who is in the room and who is not.

Engaging with decision-making systems introduces further complexity. Bridging the science-to-policy interface is widely acknowledged as a key challenge in both designing and commissioning research [47]. Barriers include capability gaps within decision-making systems or research approaches that emphasize technical refinement (e.g. modelling and data collection) over practical application. Over-reliance on this ‘knowledge-deficit model’ assumes that more evidence will automatically lead to better decisions, while ignoring contextual and political factors [10].

**Figure 1.**
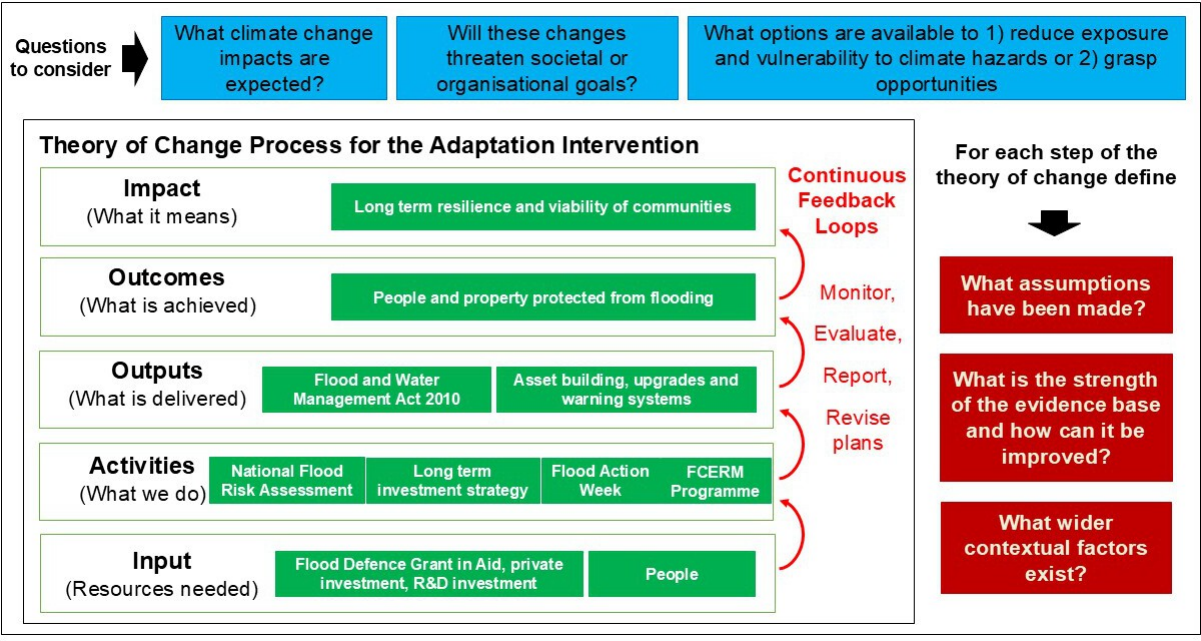
ToC model used to inform how the UK can increase resilience to flooding (adapted from the UK National Adaptation Programme [50]).

Alternatively, outcome-oriented approaches provide more actionable pathways. For instance, theory of change (ToC) models help to identify the broader system of levers (e.g. legislation, resources and standards) that influence the real-world impact of research [48]. For complex and contested issues like climate adaptation, ToC approaches benefit from systems thinking, which enables integration of multiple perspectives around shared goals [35,49]. An example of a ToC model is provided (figure 1) that illustrates the system and levers that can help increase resilience to flooding. Research is captured within the Flood and Coastal Erosion Risk Management programme (central shaded/green boxes) as an activity that helps deliver the intended outcome (figure 1).

**Figure 2.**
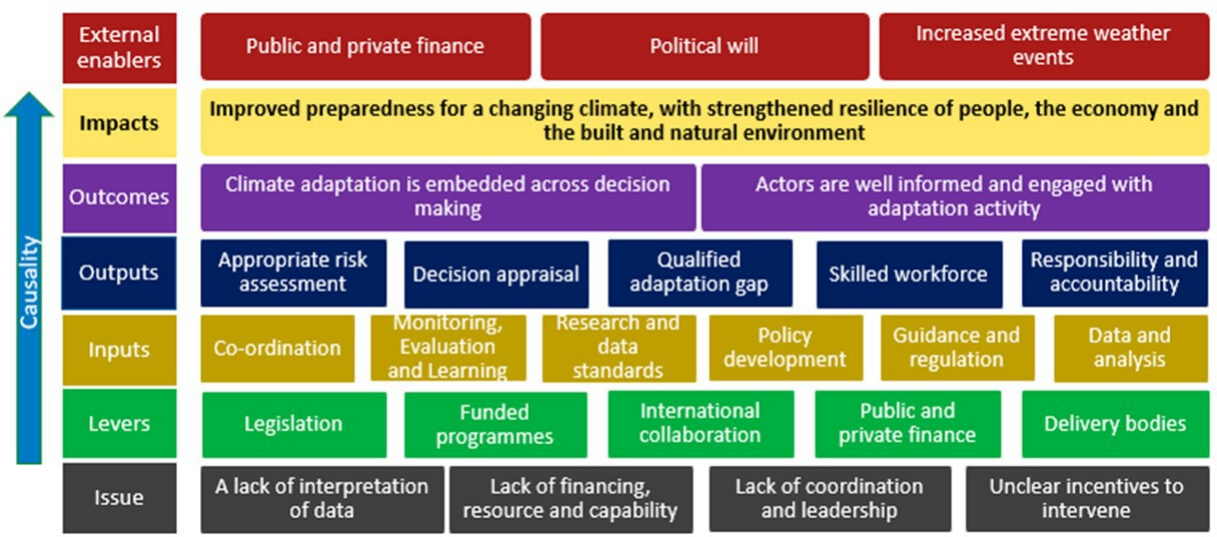
Conceptual logic model for adaptation decision-making.

Logic models are another useful tool and provide a visual representation illustrating how in theory an intervention, such as doing research, might lead to an expected outcome. A logic model developed from the perspective of a UK Government Department Adaptation Science Lead illustrates how research, data and information can be situated within broader decision-making frameworks. The model shown in figure 2 was designed to ensure that government-funded research supports societal outcomes, not just academic progress. It helps to clarify the causal links between actors, policy instruments, knowledge providers and outputs (such as guidance or risk assessments), supporting decision-makers in identifying delivery gaps.

Although ToC and logic models offer valuable framing tools, decision-making and research design typically unfold within established organizational, funding and governance structures. Pragmatic adaptations are therefore needed to contextualize research alongside competing priorities and institutional constraints. Logic models can help assess the value of existing evidence and highlight gaps, but they are rarely the sole drivers of action. External catalysts—such as political transitions or extreme weather events—can also prompt rapid shifts in decision-making that bypass formal frameworks [49].

Importantly, delivering solutions goes beyond co-design. It requires balancing research objectives with partner priorities and shaping outputs for uptake within regulatory, policy or operational contexts [44]. Impacts are not always immediate or easily measured. Although co-developed tools may be in widespread use, it is not always clear whether they have led to more resilient or equitable outcomes. Some interventions, such as the Keeping Rivers Cool programme (see §4.b), offer enduring value through community ownership and policy alignment. Others illustrate that impact often depends on iterative, relationship-based processes, not just technical outputs. These examples reinforce that success is shaped not only by the quality of evidence but by co-ownership, timing and adaptability to emergent needs.

### (b) Case studies in participatory hydrological research

While strategic information provides a critical foundation for understanding broad trends and long-term goals, effective interventions often hinge on the ability to adapt to the unique characteristics of specific contexts. In hydrology, this means aligning solutions with local hydrological conditions, community needs and socio-political realities [51–53]. For example, addressing flood risk in the UK requires not only leveraging predictive models and climate projections but also incorporating local knowledge, such as community flood histories [54,55] and existing infrastructure vulnerabilities. Practical, context-sensitive actions can address the immediate challenges while supporting broader strategic objectives [56]. This dual focus ensures that interventions are both actionable and relevant, thereby fostering community buy-in and long-term success [57].

We have collected case study examples based on our experience of work in this space. We present two examples of UK water research that highlight the importance of strategic country-scale information: Flood Allowances for Climate Change and Future Drainage. We then present two England-focused examples: Keeping Rivers Cool and weather-driven incidents to show the importance of investing time in understanding the ‘problem space’.

*Flood Allowances for Climate Change* were first formalized within the UK Planning Policy Statement 25 [58] as a top-down initiative led by regulators to guide future flood risk planning based on climate science. Required for all planning developments, these allowances describe the uplift in flood peaks as a result of climate change and include look-up tables available via Defra’s Data Services Platform [59]. Derived from national-scale modelling commissioned by regulators and developed by expert organizations, the data support planners in making informed decisions. Guidance, co-designed by operational staff and research scientists within a boundary organization (the Environment Agency), has been refined through feedback from planners and Environment Agency staff, who also deliver flood defence schemes. Although the allowances are widely used, the underlying datasets offer more value than currently realized. Complexity and uncertainty in the data may limit their use, suggesting the need for translational research and step-by-step guidance to support more nuanced applications. For example, spatial variability in flood peak sensitivity could enhance local risk assessments. As demand grows for more localized climate information, guidance has evolved but current planning rules apply mainly to new developments, offering limited support for existing infrastructure.

*Future Drainage* was co-designed to provide tailored climate data for diverse drainage design scenarios across the UK. The project emerged from industry-, consultant- and regulator-led evaluations of drainage practices and was driven by the need to understand how extreme rainfall might affect systems following the 2018 UK Climate Projections. The analyses built on earlier work by an academic-consultant team for UK Water Industry Research [60,61], using analogues, scaling relationships and a 1.5 km UK convection-permitting climate model [62]. This research programme brought together expertise from water companies, the Environment Agency, the Scottish Environment Protection Agency (SEPA) and consultants through in-person and online workshops. Together, they identified relevant parameters and timeframes, shaping outputs for immediate integration into guidance. Although the resulting 2.2 km high-resolution rainfall projections were complex and spatially variable [21], aggregating the data geographically provided a pragmatic format, enabling rapid uptake by regulators.

*The Keeping Rivers Cool programme* was a bottom-up, action-oriented initiative to help freshwaters adapt to climate change. It emerged from consensus among nature bodies that climate uncertainties would not be resolved quickly. Early researcher–practitioner collaborations advanced understanding of hydrological controls [63] and identified water temperature ‘hot spots’ [64]. Riparian tree planting was identified as a low-regret, beneficial intervention. The partnership was initially led by the Environment Agency, alongside the Forestry Commission, Natural England and the Woodland Trust. Pilot sites enabled learning-by-doing. Researchers contributed data, maps and reviews in response to emerging questions. From the outset, materials were designed to be accessible to those implementing the planting. This required licensing and information technology expertise to ensure usability on typical home computers, with boundary organizations like the Association of Rivers Trusts playing key roles. Ongoing support includes new Forestry Commission datasets that identify unshaded river reaches suitable for planting. Water temperature was also reported as a climate change indicator for the first time in 2021 [2].

*Changing nature of flood and drought incidents*. The Environment Agency is responsible for responding to floods, droughts and incidents such as pollutant spills. An embedded researcher examined the links between hydrological drivers and incident records. Such knowledge exchange roles can result in more usable products by helping researchers better understand the institutional context and co-develop tools fit for purpose [65]. In this case, the relationship between hydroclimate and incident scale proved to be nonlinear, limiting predictive potential. However, the work acted as a catalyst, leading to more participatory approaches to exploring climate change implications for incident response services [29].

## 5. Overcoming barriers and building capacity for solutions

Here we assert that bridging the gap between hydrological research and action requires investment in learning new approaches and honing skills in engagement, participation, communication and translation that serve all of society. We also need to shape enabling environments to better connect science to practice.

### (a) Building engagement, communication and translation skills

Principles for action-oriented adaptation research [66] and best practices in co-creation offer useful guidance, step-by-step methods and illustrative examples [67]. These approaches require time as well as pragmatism to maximize the use of available resources.

Hydrologists need to recognize that generating new scientific knowledge is sometimes less critical than translating existing insights into actionable knowledge through co-creation. Delivering solutions often means working with incomplete knowledge and imperfect tools. Even advanced hydrological models and forecasts cannot fully predict extreme events or specific climate outcomes (e.g. [68]). Yet, decision-makers often rely on these tools to act. Developing accessible, relevant narratives can help the application of existing knowledge, support the use of tools and avoid decision paralysis from large uncertainties in potential outcomes. The challenge lies in how researchers communicate these uncertainties while still enabling effective decision-making. The implementation of ‘rules of thumb’ or practical tools, such as those used for managing river temperatures or providing look-up tables of climate change allowances for flood risk, illustrates how imperfect science can still guide practical actions. By working with practitioners and demonstrating the potential to guide actions, researchers can provide decision-makers with actionable insights.

Communicating uncertainty effectively requires strong audience-specific communication skills and the development of relatable narratives. Strengthening communication and engagement skills, especially in participatory research and co-creation, can bridge the gap between science and decisions. This not only makes complex science more accessible to non-experts but also helps researchers better understand and integrate lived experience into the co-creation of relevant, actionable solutions. However, we must be cautious not to co-opt the lived experiences of others or assume scientific research alone can ‘fix’ everything.

### (b) Putting skills into practice fairly

Climate change affects society unequally, and without deliberate prioritization, research-driven solutions can disproportionately benefit certain sectors or groups, risking the exclusion of vulnerable communities and exacerbating social inequities. For example, climate risk maps may aid property buyers, banks and insurers, but they can also hinder homeowners facing recurrent flooding by limiting their access to insurance, reducing property values or lengthening times to sell. However, climate gentrification can occur where adaptation actions taken by wealthier populations inadvertently displace or harm more vulnerable groups [69]. Research can sometimes perpetuate existing inequities by reinforcing knowledge gaps, focusing on areas with abundant data while leaving less-resourced regions or populations unexamined [70].

Fair research requires the thoughtful building of partnerships, with attention to who is affected by specific actions and who stands to gain from them [71]. Emphasizing the dynamic interactions between water and society and advocating for the integration of human behaviours, cultural practices and societal responses into hydrological modelling can help [72]. Instead of striving solely to enhance model accuracy, we could better integrate lived experiences and qualitative insights into narratives and outcomes, to provide a richer context to model outputs, better capturing the realities affecting societal actors. For example, Coletta *et al*. [40] explored a participatory socio-hydrological modelling approach using system dynamics in Thamesmead, London, which integrated scientific expertise with stakeholder knowledge to reveal how flood resilience strategies can address the interconnected nature of urban systems.

By combining quantitative data with qualitative insights, models can better capture the complexities of human–water interactions, leading to more effective and equitable water management strategies. Tools and approaches exist to help us map the landscape of actors: their relationships, roles and spheres of influence. Tools such as ToC [73] and systems-based iterative methods [74] can help clarify how hydrological knowledge interacts with social, political and institutional contexts. System Dynamics Modelling (SDM) that combines qualitative and conceptual with quantitative and numerical approaches can enable a more complete understanding of how hydrological and other systems interconnect. Participatory SDM can help to create consensus and shared visions and has been applied in a range of environmental and water resource settings (e.g. [41]). Making greater use of lay knowledge in understanding how systems work may also improve modelling as well as avoid research outcomes that fail to resonate with decision-makers or others who are expected to respond. Engaging with communities that may be most affected by climate change requires recognition that they may also have the least social and economic capital to either participate or adapt. Nevertheless, identifying marginalized groups is critical and underscores the need to expand solution delivery to consider a broad range of stakeholder needs and interests [75].

### (c) Focusing institutional support

Translating hydrological research into societal applications requires more than simply communicating results—it depends on sustained, collaborative processes that build trust, support mutual learning and enable shared decision-making. While the emphasis on research impact is growing, many knowledge exchange efforts still follow a dissemination model, with engagement occurring only at the end of a project. A shift towards participatory and transdisciplinary approaches requires institutions to embed support for co-creation across the entire research cycle, not just at the conclusion. This means building a culture of engagement as a core activity within research communities.

The way a range of institutions, whether research employers, funding organizations or decision-making bodies, function will play a critical role in enabling this shift. Knowledge exchange fellowships, embedded researcher roles and policy placements are proven mechanisms for strengthening relationships between researchers, policymakers, practitioners and communities. Yet individuals in these bridging roles often face systemic pressures—such as the need to meet conventional academic performance metrics—that limit their ability to prioritize engagement and knowledge translation [71]. For co-creation to become a standard practice in hydrology, these roles require predictable, long-term investment.

Currently, many exchange mechanisms remain one-directional, with knowledge flowing from researchers to presumed users. Boundary organizations, knowledge brokers and science translators can help restructure these relationships by fostering shared language, agendas and understanding [17]. Crucially, institutional support for co-creation requires sufficient time, resources and recognition for iterative and nonlinear processes that extend well beyond the typical project timeframe. Initiatives such as the Flood and Drought Research Infrastructure and the Flood Hydrology Roadmap [76] offer opportunities to trial more embedded, collaborative models.

Signs of progress are emerging. The UK Research Excellence Framework has steadily increased the weight of research impact in funding assessments from 14% in 2014 to 25% in 2021. A joint United Kingdom Research and Innovation (UKRI) and Defra-funded initiative, the Maximizing Adaptation to Climate Change Hub, is supporting a transdisciplinary team of academics, practitioners and policymakers to inform UK adaptation over 3 years. Meanwhile, UK universities are being encouraged to report their engagement with non-academic partners through the Knowledge Exchange Framework (Research England).

Despite such progress, evaluating the direct influence of research on decision-making remains challenging. Science informs rather than dictates action. Still, developing metrics that track engagement, relevance and uptake can help demonstrate real-world value. Making research useful in practice requires a different kind of thinking and support: one that rewards researchers not just for what they publish, but for how they listen, adapt and co-deliver solutions. Metrics that recognize research outcomes and societal benefits, rather than focusing solely on income and citations, could help catalyse this change [77].

Co-delivering solutions in hydrology demands a careful balance of inclusivity, adaptability and collaboration. While the knowledge and tools at our disposal may be imperfect, progress is still possible through strong partnerships, transparent communication and a willingness to engage with uncertainty. By learning from past experience and committing to participatory and equitable research practices, the hydrology community can continue to advance solutions for some of the most pressing water challenges of our time.

## 6. Building a future focus for hydrological research

Climate change is accelerating beyond scientific projections. The pace and intensity of climate impacts—ranging from extreme weather events to disruptions of the global water cycle—are outpacing even the most urgent warnings from the scientific community. Global inertia on mitigation has left adaptation as the most urgent imperative.

A reorientation of hydrological research towards empowering decision-makers to adapt effectively can happen if we work differently to combat ideological, institutional and resource constraints. We can do this by:

— Building relationships with other scientists and decision-makers in government, industry and elsewhere. This takes time and involves more than asking for support in the run-up to grant applications.
— Recognizing that forming and maintaining effective relationships requires time spent in engagement activities that may not be considered ‘research’.
— Being effective research partners who recognize that others define problems differently, have timing and remit constraints and possess different types of knowledge.
— Engaging in co-creation of knowledge as an equal partner because in this way growing adaptive capacity outside research communities enhances societal benefits.
— Demanding and shaping institutional and research funding mechanisms and facilitating fluid boundaries so that participation is enabled not just for researchers but also for decision-makers.

## Data Availability

This article has no additional data.
